# Declined Activities of Daily Living as Immobility Syndrome in an Uninfected Elderly Patient Amidst a Cluster of COVID-19 Infections

**DOI:** 10.7759/cureus.17677

**Published:** 2021-09-03

**Authors:** Yoshinori Maki, Misuzu Kobayashi, Masami Kusumoto, Junichi Katsura, Ken Yanagibashi

**Affiliations:** 1 Rehabilitation, Hikari Hospital, Otsu, JPN; 2 Neurosurgery, Biwako Ohashi Hospital, Otsu, JPN

**Keywords:** geriatrics, rehabilitation, covid-19, functional independence measure, barthel index

## Abstract

Although coronavirus disease 2019 can cause immobility syndrome in the patients positive for coronavirus disease 2019 and the survivors of coronavirus disease 2019, it is not described in elderly patients who are not positive for coronavirus disease 2019.

A 90-year-old female with motor weakness of the lower extremities was admitted in the chronic care hospital. She had rehabilitation therapy for independent ambulation and toilet activities. Though she complained of irregular pulse related to atrial fibrillation during rehabilitation therapy, the symptom disappeared within a few minutes. Her ambulation and toilet activities became better with rehabilitation therapy. However, three weeks after initiating rehabilitation therapy, a cluster of coronavirus disease 2019 infections occurred. The patient was not infected, but rehabilitation therapy was discontinued for 25 days. The patient exhibited ambulation difficulty with shortness of breath and persistent irregular pulse, especially when rehabilitation therapy was reinitiated. These symptoms did not appear while the patient was outside of rehabilitation therapy. At the time of writing, she started to recover her declined ambulation with rehabilitation therapy, but was still dependent in toileting.

Immobility syndrome in elderly patients negative for coronavirus disease 2019 can be aggravated following the occurrence of a low-level cluster of coronavirus disease 2019 infections. Medical workers should, therefore, always consider this hidden risk and should plan an adequate program in the early period of rehabilitation therapy for the elderly patients.

## Introduction

The coronavirus disease 2019 (COVID-19) pandemic, caused by severe acute respiratory syndrome coronavirus 2, has become a global health issue [1-4], with clinical symptoms varying from mild to severe. Patients with COVID-19 who manifest severe symptoms require hospitalization, oxygen therapy, and admission to the intensive care unit [5]. The sequelae of COVID-19 include fatigue, breathlessness, joint pain, muscle weakness, and functional impairment [3,4].

Rehabilitation management for patients and survivors of COVID-19 has also been reported. The significance of rehabilitation therapy during the recovery phase of COVID-19 is as important as that of rehabilitation therapy during the acute phase [1-7]; however, the influence of the COVID-19 pandemic on immobility syndrome in elderly individuals without COVID-19 has not been well described. Here, we present the case of an elderly patient without COVID-19 infection in which immobility syndrome was aggravated due to the suspension of rehabilitation therapy following the occurrence of clustered COVID-19 infections.

## Case presentation

A 90-year-old female was admitted to the chronic care hospital. The patient lived with her husband and was independent regarding cooking and feeding. She had a history of fracture of the right ankle for which she underwent an operation six years before. Though muscle of bilateral lower extremities was gradually getting weak since this episode, her indoor ambulation was sufficiently independent with the aid of a rehabilitation walker. However, she required support from either her husband or a wheelchair for ambulation at home due to the pain in the right knee related to gonarthrosis. She did not take any analgesic for the pain in the right knee. The patient had fallen while moving from a toilet to a wheelchair; thereafter, she was unable to transfer herself to the toilet due to anxiety about a repeated falling episode. As her ability to transfer to the toilet declined, incontinence recurred. She was, therefore, admitted to the hospital for rehabilitation therapy to regain independence regarding toileting and indoor ambulation. The patient had anemia, atrial fibrillation, and stomach cancer in the early stage. As the patient did not wish any treatment for the stomach cancer, the stomach cancer was under observation. She was taking iron supplementation, lansoprazole 15 mg, and verapamil 120 mg per day. On admission, the patient was examined by both occupational and physical therapists. Her height was 155 cm, and her body weight was 56 kg (body mass index = 23.3). She could not extend fully bilateral knees due to pain and muscle weakness and her back was kyphotic. However, the patient could stand still with that posture. She was able to walk only for approximately 6 m on parallel bars. After excretion in toilet, she could not lift up the slacks to the west. She had a score of 30 on the revised Hasegawa’s Dementia Scale, while the initial functional independence measure (FIM), motor, and cognitive subtotal scores were 59, 32, and 27 respectively (Table 1); her Barthel Index (BI) on admission was 45. The scores regarding ambulation, grooming, and toilet activities were severely reduced (Table 2). Rehabilitation therapy for ambulation and independence of toileting was initiated. A physical therapist trained the patient 60 minutes a day and an occupational therapist trained her 40 minutes a day. Rehabilitation therapy was performed four to five days a week. During rehabilitation therapy, the patient needed rest due to irregular pulse, but the symptom disappeared within a few minutes. Three weeks after the initiation of rehabilitation therapy, the patient was able to walk for approximately 24 m on parallel bars. As the standing position of the patient started to be stable, the patient was able to walk for several meters with the aid of a rehabilitation walker. She started also to succeed to lift the slacks to the west after excretion in toilet. The vital signs of the patient were almost stable as systolic blood pressure of 95-110 mmHg and pulse of 70-80 per minute. However, a cluster of COVID-19 infections occurred at the hospital. All infected patients were transferred to other hospitals, and although the patient was not infected, rehabilitation therapy was ceased under the public health administration to avoid the risk of spreading COVID-19 through rehabilitation therapists. During the period of therapy suspension, the patient could only transfer to the toilet and dining hall with the aid of a wheelchair, while continuing herself to exercise by lifting bilateral legs while lying in the bed several times a day. During the cessation period of rehabilitation therapy, the patient started complaining of muscle weakness of the lower extremities. Thus, the patient required a flip-up wheelchair when she moved from the bed. Rehabilitation therapy was resumed two weeks after the last COVID-19-positive patient was identified; therefore, she did not receive rehabilitation therapy for a total of 25 days. The patient was re-evaluated when rehabilitation therapy was reinitiated and FIM and BI were assessed. The patient was not able to stand stably. The patient was able to walk for less than 6 m on parallel bars. The patient reported lethargy during therapy, and required additional support to get up from the sitting position; she also mentioned shortness of breath and an irregular pulse during toilet activities. When she complained of these symptoms, systolic blood pressure was 110-120 mmHg and pulse was 79-106 per minute. These symptoms did not disappear for more than 5 minutes even with suspension of rehabilitation therapy, which was not observed in the patient before the cluster of COVID-19 infections had occurred. However, the patient did not complain of lethargy outside of rehabilitation therapy. The FIM, and motor and cognitive subtotal scores were 54, 29, and 25, respectively (Table 1); the BI score was 40 (Table 2). The transfer scores decreased in both assessments, while the scores for comprehension and memory in the FIM also slightly decreased. At the time of writing, the patient continued to undergo rehabilitation therapy. The patient demonstrated recovery and was able to walk for 21 m with gait training on parallel bars, three weeks after reinitiating rehabilitation therapy. Shortness of breath or any irregular pulse did not last for more than 5 minutes as the patient's performance ameliorated with rehabilitation therapy. However, she did not achieve the goal of rehabilitation therapy to be independent of toileting.

**Table 1:** Functional independence measure scores on admission and after the occurrence of the COVID-19 infection cluster COVID-19, coronavirus disease 2019.

	On Admission	After Occurrence of the COVID-19 Infection Cluster
Self-Care		
Eating	7	7
Grooming	5	5
Bathing	1	1
Dressing (Upper Body)	1	1
Dressing (Lower Body)	1	1
Toileting	1	1
Sphincter Control		
Bladder Management	2	2
Bowel Management	2	2
Transfers		
Bed, Chair, Wheelchair	4	2
Toilet	4	3
Tub, Shower	1	1
Locomotion		
Walk (W)/Wheelchair (C) /Both(B)	2 C	2 C
Stairs	1	1
Motor Subtotal Score	32	29
Communication		
Comprehension	5	4
Expression	5	5
Social Cognition		
Social Interaction	7	7
Problem Solving	5	5
Memory	5	4
Cognitive Subtotal Score	27	25
Total FIM Score	59	54

**Table 2:** Barthel Index scores on admission and after the occurrence of the COVID-19 infection cluster COVID-19, coronavirus disease 2019.

	On Admission	After Occurrence of the COVID-19 Infection Cluster
Feeding	10	10
Bathing	5	5
Grooming	0	0
Dressing	5	5
Bowels	5	5
Bladder	5	5
Toilet use	5	5
Transfer (Bed to Chair and Back)	10	5
Mobility (On Level Surfaces)	0	0
Stairs	0	0
Total Score	45	40

## Discussion

Here, we present the case of an elderly patient with worsened immobility syndrome following the suspension of rehabilitation therapy during the occurrence of clustered COVID-19 infections in the chronic care hospital. Although the patient was not infected, rehabilitation therapy was suspended to prevent the spread of COVID-19. As a result of worsened immobility syndrome, the patient’s transfer ability weakened, while her cognitive ability slightly decreased. Clinical symptoms, such as shortness of breath and persistent irregular pulse, which were not observed on admission, appeared when rehabilitation therapy was reintroduced.

Immobility syndrome during hospitalization in elderly patients is a clinically important concern, as activities of daily living and ambulatory function tend to decline during hospitalization [8]. The musculoskeletal, circulatory, and respiratory systems, as well as cognitive function can be impaired due to immobility syndrome; therefore, clinical symptoms related to immobility syndrome can vary, and may include muscle weakness, joint rigidity, osteoporosis, decreased function of the heart and lungs, deep vein thrombosis, and cognitive impairment [9]. Our patient was evaluated using the FIM and BI during hospitalization in the hospital. She had already been suffering from limited activities of daily living due to muscle weakness of bilateral lower extremities and the pain of the right knee. Even after undergoing three weeks of rehabilitation therapy, her transfer ability declined after rehabilitation was suspended. 

Because the patient had already medication for anemia and atrial fibrillation, irregular pulse manifested in the patient prior to the cluster of COVID-19 infections in the hospital. However, the symptoms resolved with a few minutes of rest. After suspension of rehabilitation therapy, irregular pulse became slightly persistent and shortness of breath also appeared. As the patient was hospitalized in the chronic care hospital where specialists of internal medicine such as cardiologist and pulmonologist did not work, her cardiac and pulmonary functions were not examined. However, the patient was not complaining of lethargy while she was outside of rehabilitation therapy. Thus, we thought that persistent irregular pulse and shortness of breath could be induced by exercise load of rehabilitation therapy, not by worsened cardiac or pulmonary function. Her respiratory and circulatory symptoms may have been a result of the immobility syndrome. Concerning mobilization ability of the patient, decline of transfer ability already manifested during the period of the cluster of COVID-19 infections; the patient needed a flip-up wheelchair, which was not necessary when the cluster of COVID-19 infections had occurred. The distance for which the patient was able to walk on parallel bars after the cluster of COVID-19 infections apparently reduced in comparison with that prior to the cluster of COVID-19 infections. The patient was able to walk for longer distance on parallel bars three weeks after reinitiating rehabilitation therapy. Hence, we considered also that the declined transfer ability of the patient could result from immobility syndrome. While rehabilitation therapy was suspended, the patient exercised herself by lifting her legs on the bed twice to thrice per day; however, this simple exercise was not enough to prevent the aggravation of immobility syndrome. As the patient marked a score of 30 on the revised Hasegawa’s Dementia Scale, introduction of an independent program in the early period of the first session of rehabilitation therapy could have been effective and helpful to prevent immobility syndrome. In that case, decline of transfer ability of the patient could have been slight.

The strategy and effectiveness of rehabilitation management focuses on the patients and survivors of COVID-19 [1-7]. Various means of remote rehabilitation training, based on telehealth technology such as wearable devices, mobile phone applications, virtual reality, and robots, may be an ideal option for patients who could not undergo rehabilitation therapy due to the cluster of COVID-19 infections [5,7,10]. Still, the feasibility of telerehabilitation therapy may not be suitable for elderly patients who are unfamiliar with the use of telehealth technology; thus, the invention of simple programs and devices applicable in the chronic care hospital seems also expected if telerehabilitation therapy is considered in elderly patients.

Medical workers should always consider the possible risk of immobility syndrome, which can aggravate the abilities of elderly patients not infected with COVID-19 amidst the pandemic. In this paper, we present a single, representative case of immobility syndrome caused by suspension of rehabilitation therapy due to the occurrence of a cluster of COVID-19 infections in the hospital. We are currently collecting the clinical data of elderly patients hospitalized during the period of suspended rehabilitation therapy; we, thus, aim to successively report the impact of immobility syndrome in these patients regarding the occurrence of a cluster of COVID-19 infections.

## Conclusions

Our case suggests that the elderly patients negative for COVID-19 can suffer from aggravation of immobility syndrome due to a low-level cluster of COVID-19 infections in the chronic care hospital. Rehabilitation therapist should consider always this hidden risk under the pandemic of COVID-19. Rehabilitation therapist should plan an effective independent program especially for the elderly patients without apparent cognitive impairment. 
